# The risk allele of SNP rs3803662 and the mRNA level of its closest genes *TOX3* and *LOC643714* predict adverse outcome for breast cancer patients

**DOI:** 10.1186/1471-2407-12-621

**Published:** 2012-12-27

**Authors:** Eydis Th Gudmundsdottir, Rosa B Barkardottir, Adalgeir Arason, Haukur Gunnarsson, Laufey Th Amundadottir, Bjarni A Agnarsson, Oskar Th Johannsson, Inga Reynisdottir

**Affiliations:** 1 Department of Pathology, Landspitali-University Hospital, Hringbraut, 101, Reykjavik, Iceland; 2 BMC, Faculty of Medicine, University of Iceland, Vatnsmyrarvegi 16, 101, Reykjavik, Iceland; 3 Present address: Actavis, Hafnarfjordur, Iceland; 4 Department of Health and Human Services, Laboratory of Translational Genomics, Division of Cancer Epidemiology and Genetics, National Cancer Institute, National Institutes of Health, Bethesda, MD, USA; 5 Department of Oncology, 20A, Landspitali-University Hospital, Hringbraut, 101, Reykjavik, Iceland

**Keywords:** TOX3, LOC643714, rs380662, Risk allele, Breast cancer, Clinical, Pathological, Survival, Oestrogen receptor

## Abstract

**Background:**

The minor allele of SNP rs3803662 has been shown to correlate with increased breast cancer risk and with lower expression of *TOX3*. The SNP is closely located to *TOX3* residing within an uncharacterised gene *LOC643714*. The aim of the study was to examine the association of the risk allele with expression of *TOX3* and *LOC643714,* and of mRNA levels and genotype with clinical and pathological characteristics.

**Methods:**

The SNP was genotyped in DNA isolated from blood and normal tissue from 160 breast cancer patients and mRNA levels were measured by microarrays and quantitative real-time (qRT)-PCR in breast tumours. Association with clinical and pathological characteristics was analysed by parametric tests.

**Results:**

An association of the risk allele of rs3803662 with lower *TOX3* expression was confirmed in oestrogen receptor (ER) positive tumours. It was more often observed in lobular tumours (p = 0.04), and carriers of the risk allele who had been diagnosed with luminal A tumours had shorter overall survival (OS) than carriers of the non-risk allele (p = 0.01). Positive correlation between the mRNA levels of *TOX3* and *LOC643714* was observed (r = 0.44 and p < 0.001). Association analysis with tumour pathology showed that low *TOX3* and *LOC643714* expression correlated with high Ki67 levels (p = 0.026 and p = 0.002) and the basal subtype (p < 0.001 and p < 0.001), whereas high expression correlated with ER (p = 0.004 and p < 0.001) and progesterone receptor (PgR) (p = 0.005 and p < 0.001) expression. Furthermore, high TOX3 and LOC643714 correlated with positive lymph nodes (p < 0.001 and p = 0.01). Patients with ER positive tumours and high levels of *TOX3* mRNA had shorter overall- and distant metastasis free-survival (p = 0.017 and p = 0.021), an effect mostly attributable to patients with luminal B tumours.

**Conclusions:**

The results suggest that the effect of the risk allele of rs3803662 is strongest in luminal A tumours and that the expression levels of *TOX3* and/or *LOC643714* affect the progression of breast cancer. The effect may vary depending on the subtype and developmental stage of the tumour.

## Background

Breast cancer is the most frequent cancer in women accounting for 23% of all cancers in 2008 [1]. It shows strong familial aggregation being about twice as common in first-degree relatives of individuals with the disease than in the general population [2]. Familial linkage studies have identified high penetrance, low frequency mutations in genes such as the breast cancer susceptibility genes *BRCA1* and *BRCA2* but these mutations only account for approximately 20% of the familial risk (reviewed in [3]). In unselected breast cancer patients, genome-wide association studies have identified low penetrance, high frequency SNPs that are associated with breast cancer risk (reviewed in [4]). SNP rs3803662, at 16q12, is one of them. It was demonstrated that the minor allele of rs3803662 conferred increased risk of breast cancer in European women [5,6], a finding that has been validated in large studies of unselected patients and patients with a familial history of breast cancer and meta-analyses [7-13]. This finding was also observed in women of East Asian descent [14-16] but not in women of African American descent [17-19]. The association was confined to oestrogen receptor positive cancer [6,20] but studies in large consortia have shown associations in ER positive and negative breast cancer, albeit stronger in ER positive disease [21,22]. The minor rs3803662 allele also increased the risk of breast cancer in BRCA1 and BRCA2 carriers [21,23,24] as well as in a population-based study of men [25].

The genes located closest to rs3803662 are *TOX3* and *LOC643714*. The SNP is in the last exon (exon 4) of *LOC643714* and near the 5’ end of *TOX3*. The chromosomal region spanning the 5’ end of *TOX3*, the intergenic region between *TOX3* and *LOC643714* and the entire coding part of *LOC643714* are located in a 133 kb linkage disequilibrium (LD) block [13]. Other SNPs within this LD block show association with breast cancer but rs3803662 shows the strongest effect [5,13]. *LOC643714* is an uncharacterised gene whose function is unknown [26]. The *TOX3* gene, also known as *TNRC9* and *CAGF9*, has a tri-nucleotide repeat motive [27] and a high mobility group (HMG)-box [28]. It is involved in the regulation of calcium dependent transcription and interacts with cAMP-response-element-binding protein CREB and CBP (CREB-binding protein) [29]. Additionally, TOX3 can interact with CITED1 and increase transcription [29,30]. CITED1 is a transcription co-regulator that enhances the activity of transcription factors such as the ER [31] and SMAD4 [32].

A correlation of the rs3803662 genotype with lower *TOX3* mRNA has been observed in breast tumours where *TOX3* mRNA expression decreased in an allele-dependent manner [33]. So far, one study showed an effect of *TOX3* expression on breast cancer in that increased level of *TOX3* mRNA predicted breast cancer metastasis to bone [34]. In the present study we report the results of association analysis between the rs3803662 genotype and expression of its closest genes, *TOX3* and *LOC643714,* in a cohort of breast cancer patients as well as the correlation analyses between rs3803662, and expression of *TOX3* and *LOC643714*, with known clinical and pathological characteristics.

## Methods

### Tumour and normal tissue samples

Freshly frozen primary breast tumour samples (*n* = 160) were obtained from the Department of Pathology at Landspitali University Hospital, Reykjavik, Iceland as well as normal tissue samples embedded in paraffin if a blood sample was not available from the patient. The patients were diagnosed during the years 1987–2003. Informed consent was obtained from all participants in the study. The study was approved by the Icelandic Data Protection Authority (2001/523 and 2002/463) as well as the National Bioethics Committee of Iceland (99/051 and 99/051_FS1) and a written consent was obtained from the participants in the study according to national guidelines.

### DNA isolation and SNP genotyping

DNA isolation from blood samples was performed according to Miller et al. [35], and from paraffin-embedded normal tissue using a xylene treatment followed by proteinase K digestion and phenol/chloroform/isoamyl alcohol purification. Taqman assay C_25968567_10 (Applied Biosystems) was used to genotype SNP rs3803662 using 20 ng of genomic DNA isolated from the blood samples and the normal tissue. The reaction was carried out in StepOne Real-Time PCR System (Applied Biosystems). The allele frequencies of rs3803662 were in Hardy-Weinberg Equilibrium (p > 0.1).

### RNA isolation and quantification

The microarray gene expression data for *TOX3* were retrieved from a study including 359 breast tumours [36] via GEO (GSE22133 [37]). qRT-PCR was used to confirm the array data mRNA levels of *TOX3* and *LOC643714* in total RNA that was isolated from the identical fresh frozen breast tumours which were used in the first study. RNA was isolated using Trizol (Invitrogen) followed by RNeasy Midi Purification Kit (Qiagen) according to the manufacturer protocol. RNA (2 μg) was reverse transcribed with random hexamer primers using the RevertAid Minus First strand cDNA synthesis Kit (Fermentas). Quantification of gene expression was performed with Taqman Gene Expression Assays (Applied Biosystems) using 1 and 200 ng cDNA as template for *TOX3* (Hs01101330_m1) and *LOC643714* (Hs01000553_m1), respectively. Forty rounds of amplification were performed for *TOX3* and 50 rounds for *LOC643714*. The gene expression was calculated relative to the expression of the reference gene TATA-binding protein (*TBP*, 4326322E Applied Biosystems) according to the formula: mRNA expression of target gene = 2^-(mean Ct target – mean Ct reference)^. Expression was measured on available RNA samples; 139 and 126 RNA samples for *TOX3* and *LOC643714*, respectively. The level of mRNA expression of *LOC643714* was much lower in all samples tested and in 30 of the 126 samples *LOC643714* mRNA was below the range detectable by qRT-PCR. To include these samples in the association studies the samples were given a Ct value that was 3.3 cycles higher than the highest *LOC643714* Ct value measured. This calculated Ct value was used to calculate the level of *LOC643714* mRNA expression in the 30 tumours relative to the expression of the reference gene *TBP* according to the formula given above.

### Clinical information and tumour characteristics

Information regarding the date and cause of death was obtained from patient records and confirmed by the Icelandic National Register. The age at diagnosis and information about tumour size, type and nodal status as well as ER, progesterone receptor (PgR), ErbB2 and Ki67 levels, was retrieved from records at the Department of Pathology at the Landspitali-University Hospital. ER, PgR, ErbB2 and Ki67 levels were evaluated by immunohistochemical staining according to the manufacturer’s protocol (Dako): ER (M7047, clone 1D5, 1:100), PgR (M3569, clone PgR 636, 1:100), HercepTest (K5207), and Ki67 (M7240, clone MIB1, 1:100). The ER and PgR status was determined as being either positive (staining 1+ to 3+) or negative (no staining). The ErbB2 receptor status was determined as being either positive (3+ staining plus 2+ staining and positive FISH analysis) or negative (1+ plus 2+ staining and negative FISH analysis) [38]. Tumours staining for Ki67 were scored highly proliferative if the number of cells and staining intensity was above 10% and slowly proliferating below 10%. The tumours were categorized according to the WHO histological classification and the histological grade was determined by the modified Bloom-Richardson system. The molecular subtypes of the tumours were retrieved from GEO dataset GSE22133 [37].

### Statistical analysis

Pearson’s χ^2 ^analysis was used to test genotype frequencies for deviations from Hardy-Weinberg equilibrium (HWE) for SNP rs3803662. Fisher exact test was used to analyse association between the SNP genotype and clinical and pathological parameters. The association of the SNP genotype and mRNA expression was determined using ANOVA. The association of mRNA expression, as measured by qRT-PCR, to clinical and pathological parameters was determined using a t-test or ANOVA. Pearson correlation coefficient was calculated to determine the strength of the linear relationship between *TOX3* and *LOC643714* mRNA expression. Analyses of overall survival (OS) and distant metastasis-free survival (DMFS) were calculated by using the Kaplan-Meier method and the log-rank test. All calculations were performed using the statistical program R [39] version 2.11.1. Schematic of the analyses and significant results are depicted for the SNP and mRNA in Additional file 1: Figures S5 and S6, respectively.

## Results

### The rs3803662 risk allele is associated with shorter OS

**Figure 1:**
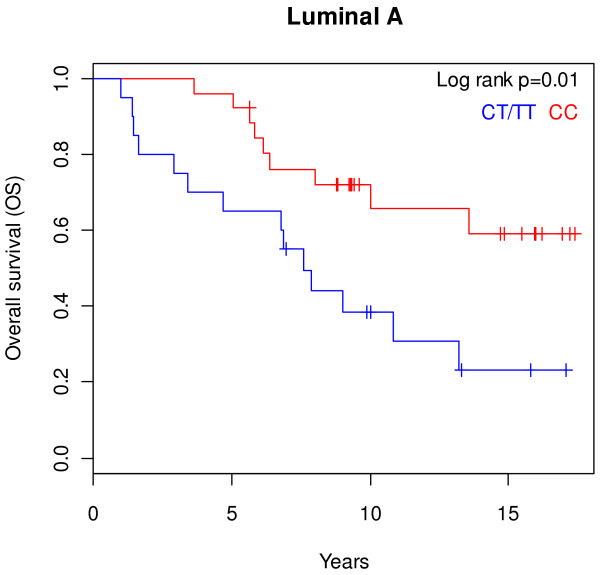
**Patients with luminal A tumours and the risk rs3803662 allele have shorter OS.** The OS was compared in patients with luminal A tumours who were homo- or heterozygous for the risk rs3803662 allele (CT/TT, n = 20) as opposed to homozygous for the non-risk rs3803662 allele (CC, n = 26), log rank p = 0.01.

Correlation analysis between the SNP genotypes and clinical and pathological factors of breast cancer was performed. The comparison was made between patients who were homo- or heterozygous for the risk allele and patients homozygous for the non-risk allele. We observed a significant association between the risk allele and lobular tumours (p = 0.04; Additional file 2: Table S1). An association was detected between survival and the risk allele in the entire cohort. However, when survival was analysed according to molecular subtypes, patients diagnosed with luminal A tumours who carried the risk allele had shorter OS as compared to patients homozygous for the non-risk allele (p = 0.01, Figure 1). Distant metastasis-free survival (DMFS) was also shorter in this patient group but the difference did not reach significance (p = 0.24, Additional file 1: Figure S1). The rs3803662 genotype did not show correlation with OS or DMFS in other breast cancer subtypes.

### rs3803662 is associated with decreased *TOX3* mRNA expression in ER positive tumours

**Figure 2:**
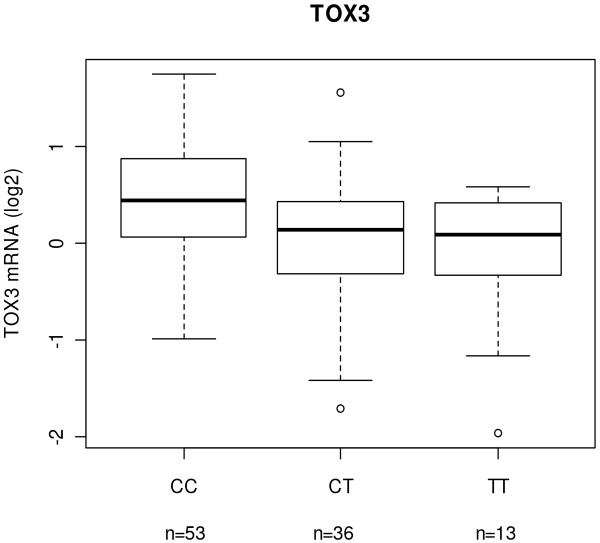
**The minor allele of rs3803662 is associated with lower *TOX3 *mRNA expression.** Correlation of mRNA expression level of *TOX3* with genotype of rs3803662 in 102 ER positive breast cancer tumours (p = 0.002). The mRNA levels were measured on microarrays. ANOVA was used to test for significant difference between the three genotype groups.

Gene expression data from microarrays were available for *TOX3* in 160 breast tumours. Expression of *TOX3* mRNA was compared to the rs3803662 genotype in the total set of tumours as well as in ER positive and ER negative tumours. In ER positive tumours, *TOX3* expression decreased significantly with the number of risk alleles (p = 0.002, Figure 2). No difference was seen in ER negative or the entire group of tumours (p = 0.25 and p = 0.29, respectively). To confirm these results, qRT-PCR was performed to measure *TOX3* mRNA levels in 139 breast tumours available from the microarray study. The qRT-PCR results correlated positively with the microarray results (r = 0.57, p < 0.001) and showed that the *TOX3* mRNA level decreased with the number of risk alleles in ER positive tumours although the difference did not reach significance (p = 0.11, Additional file 1: Figure S2A). As before, no difference was observed in the ER negative or entire group of tumours (p = 0.78 and p = 0.53, respectively). Due to lack of microarray expression data of *LOC643714* qRT-PCR was performed to measure the mRNA levels. There was correlation between the mRNA levels of *TOX3* and *LOC643714* as measured by qRT-PCR (r= 0.45, p < 0.001). Similar to *TOX3,* the mRNA levels of *LOC643714* decreased with the number of risk alleles in ER positive tumours but the difference did not reach significance (p = 0.24, Additional file 1: Figure S2B). The majority of ER positive tumours are of the molecular subtypes luminal A and luminal B. Although not significant, a lower expression of *TOX3* and *LOC643714* was seen in tumours with the risk allele in luminal A tumours but not in luminal B tumours (Additional file 1: Figure S3).

### High *TOX3* and *LOC643714* mRNA correlated with positive lymph nodes and ER positive tumours

**Figure 3:**
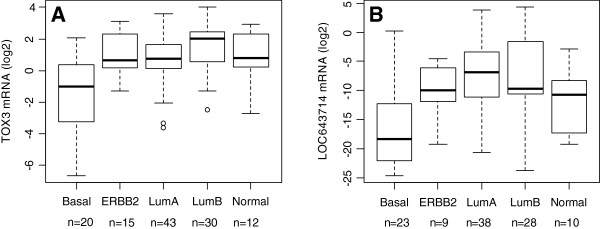
***TOX3 *and *LOC643714 *mRNA expression was lowest in tumours of the basal subtype.** The mRNA expression levels in: **A**) *TOX3* (p < 0.001) and **B**) *LOC643714* (p < 0.001) in five breast tumour subtypes. ANOVA was used to test for differences in expression between the different subtypes. The difference in *TOX3* expression between basal and ERBB2 tumours was significant (p = 0.02).

Expression levels of *TOX3* and *LOC643714* were significantly higher in ER positive tumours than in ER negative tumours (p = 0.004 and p < 0.001, respectively; Table 1). PgR positive tumours also had significantly higher *TOX3* and *LOC643714* mRNA levels than PgR negative ones (p = 0.005 and p < 0.001, respectively). This was reflected in different mRNA levels of the two genes according to molecular subtypes. Luminal A and B tumours, that are mainly ER positive, expressed significantly higher mRNA than ER negative basal tumours (*TOX3* and *LOC653714* mRNA vs. subtypes: p < 0.001 and p < 0.001, respectively, Table 1 and Figure 3). Interestingly, two-thirds of the ERBB2 tumours were ER negative, yet they expressed significantly higher *TOX3* mRNA than basal tumours (p = 0.02, Figure 3). Tumours of patients who were lymph node positive when diagnosed, had significantly higher *TOX3* and *LOC643714* mRNA levels than tumours of lymph node negative patients (p < 0.001 and p = 0.04, respectively, Table 1). Tumours with high expression of Ki67 had significantly lower *TOX3* and *LOC643714* mRNA levels than tumours with low expression of Ki67 (p = 0.026 and p < 0.001, respectively). Furthermore, low mRNA level of *LOC643714* associated with a high histograde (p = 0.01) which likely echoed the strong correlation of basal tumours with high histograde (data not shown). ErbB2 negative tumours expressed more *LOC643714* mRNA than ErbB2 positive tumours (p = 0.04), which was not unexpected because the majority of the ErbB2 negative tumours was ER positive.

**Table 1:** ***TOX3 *and *LOC643714 *mRNA expression compared with clinical and pathological characteristics**

		**n**	***TOX3 *mRNA**	**p-value**	**n**	***LOC643714 *mRNA**	**p-value**
		*139*	median (25% and 75%)		*126*	median (25% and 75%)	
**Age**							
	< 50	59 (42%)	1.92 (0.9 - 4.5)	0.89	53 (42%)	3.1 E-4 (5.6 E-6 - 1.5 E-2)	0.07
	≥ 50	80 (58%)	1.68 (1.1 - 4.0)		73 (58%)	1.7 E-3 (1.1 E-4 - 8.5 E-2)	
**Tumour size (mm)**							
	≤ 20	42 (31%)	1.5 (1.0 - 4.8)	0.45	39 (31%)	6.4 E-4 (2.7 E-5 - 1.0 E-2)	0.26
	> 20	95 (69%)	2.0 (1.0 - 4.5)		85 (69%)	1.1 E-3 (9.2 E-5 - 8.5 E-2)	
	unknown	2			2		
**Estrogen receptor**							
	negative	44 (33%)	1.3 (0.4 - 3.3)	0.004	41 (34%)	9.3 E-6 (6.4 E-7 - 5.5 E-4)	< 0.001
	positive	91 (67%)	2.1 (1.1 - 4.5)		81 (66%)	2.4 E-3 (4.6 E-4 - 9.8 E-2)	
	unknown	4			4		
**Progesterone receptor**							
	negative	65 (49%)	1.4 (0.6 - 4.2)	0.005	59 (50%)	1.9 E-4 (1.4 E-6 - 3.1 E-3)	< 0.001
	positive	67 (51%)	2.1 (1.3 - 4.3)		60 (50%)	3.3 E-3 (4.2 E-4 - 0.5)	
	unknown	7			7		
**ErbB2 status**							
	negative	114 (83%)	1.8 (1.0 - 4.2)	0.81	107 (86%)	1.1 E-3 (6.6 E-5 - 7.7 E-2)	0.04
	positive	24 (17%)	1.9 (0.8 - 5.0)		18 (14%)	2.7 E-4 (4.7 E-5 - 9.2 E-4)	
	unknown	1			1		
**Nodal status**							
	negative	56 (44%)	1.3 (0.4 - 3.4)	< 0.001	53 (46%)	5.3 E-4 (1.9 E-6 - 2.3E-2)	0.04
	positive	71 (56%)	2.2 (1.2 - 5.3)		62 (54%)	1.4 E-3 (2.0 E-4 - 5.7 E-2)	
	unknown	12			11		
**Ki67**							
	Low	97 (71%)	2.1 (1.1 - 4.4)	0.026	87 (70%)	2.4 E-3 (2.2 E-4 - 9.0 E-2)	< 0.001
	High	39 (29%)	1.2 (0.7 - 2.4)		37 (30%)	9.6 E-5 (1.9 E-6 - 9.0 E-4)	
	unknown	3			2		
**Histograde**							
	1	12 (9%)	1.6 (0.8 - 3.5)	0.16	12 (9%)	1.1 E-3 (2.7 E-4 - 3.5 E-2)	0.01
	2	78 (57%)	1.9 (1.1 - 4.4)		69 (56%)	3.5 E-3 (1.9 E-4 - 9.8 E-2)	
	3	47 (34%)	1.6 (0.5 - 4.2)		43 (35%)	2.3 E-4 (3.3 E-6 - 1.5 E-3)	
	unknown	2			2		
**Familial status **^**1**^							
	BRCA2	26 (19%)	2.9 (0.8 - 5.1)	0.90	25 (20%)	6.7 E-4 (1.2 E-4 - 4.2 E-3)	0.40
	Non-BRCA2	112 (81%)	1.6 (1.0 - 4.2)		100 (80%)	1.2 E-3 (6.3 E-5 - 6.4 E-2)	
	unknown	1			1		
**Subtype**							
	Basal	20 (17%)	0.5 (0.1 – 1.3)	< 0.001	23 (21%)	3.1 E-6 (2.3 E-7 - 2.0 E-4)	< 0.001
	ERBB2	15 (12%)	1.6 (1.2 - 5.0)		9 (8%)	9.7 E-4 (2.7 E-4 - 1.5 E-2)	
	Luminal A	43 (36%)	1.6 (1.1 - 3.1)		38 (35%)	8.9 E-3 (5.0 E-4 - 9.8 E-2)	
	Luminal B	30 (25%)	4.0 (1.6 - 5.4)		28 (26%)	1.2 E-3 (6.7 E-4 – 3.0 E-1)	
	Normal-like	12 (10%)	1.8 (1.2 - 4.4)		10 (9%)	5.8 E-4 (3.4 E-5 - 2.7 E-3)	
	Unclass/unknown	19			18		
**Histological type**							
	Ductal	116 (86%)	2.1 (1.0 - 4.5)	0.12	105 (86%)	1.1 E-3 (9.2 E-5 - 5.2 E-2)	0.91
	Lobular	11 (8%)	1.3 (1.3 - 2.2)		9 (7%)	6.2 E-4 (1.2 E-4 - 7.8 E-3)	
	other	8 (6%)	1.0 (0.6 - 1.5)		8 (7%)	9.8 E-5 (5.2 E-6 - 2.2 E-2)	
	unknown	4			4		
**Metastasis**							
	negative	63 (50%)	1.6 (0.9 - 3.1)	0.18	63 (55%)	6.4 E-4 (6.3 E-6 - 1.9 E-2)	0.16
	positive	63 (50%)	1.9 (1.1 - 4.9)		52 (45%)	1.6 E-3 (1.4 E-4 - 8.1 E-2)	
	unknown	13			11		

The table shows the median and the 25th and 75th percentiles. The p-value is calculated with log_2 _transformed data using a *t-*test or ANOVA.

^1^ One tumour sample was BRCA1 positive and was not used in familial status calculations.

Due to the difference in expression of *TOX3* and *LOC643714* according to ER status, the association analysis was performed separately for the ER positive and negative subgroups. The results of the correlation analyses in the ER positive subgroup were similar to the whole group of tumours although in most cases it did not reach significance, possibly due to lower number of cases included in the analyses (Additional file 2: Table S2). The only significant correlation remaining was the one between high *LOC643714* mRNA and low Ki67 (p = 0.02) and negative ErbB2 status (p = 0.01, Additional file 2: Table S2). Expression of *LOC643714* correlated with that of *TOX3* in ER negative tumours (r = 0.64, p < 0.001) but not in ER positive tumours (r = 0.13, p = 0.25). The result for the ER negative tumour subgroup was similar to the whole group of tumours in that high *TOX3* and *LOC643714* mRNA levels were associated with positive lymph nodes (p < 0.001 and p = 0.01, respectively) and a positive PgR status, although only significant for *TOX3* (p = 0.002, Additional file 2: Table S3). But there was lack of correlation between low mRNA levels of *TOX3* and *LOC643714* and high expression of Ki67 and low mRNA level of *LOC643714* and negative ErbB2 status of the tumour as well as high histograde. A significant correlation was observed between high *TOX3* mRNA in tumours of patients with metastasis (p = 0.015, Additional file 2: Table S3), which was observed as a trend in the whole group of tumours (Table 1).

### Elevated *TOX3* mRNA expression in breast tumours is associated with poor survival

**Figure 4:**
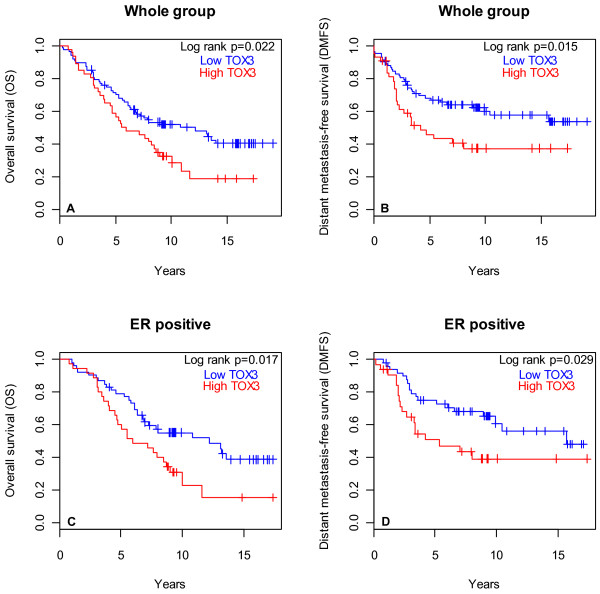
**High *TOX3 *mRNA was associated with shorter survival.** Tumours were classified into high and low expressing tumours based on mRNA levels that were above or below average, respectively. Accordingly, survival was analysed in the whole group of patients: A) OS: 88 low vs. 46 high, log rank p = 0.022; B) DMFS: 83 low vs. 43 high, log rank p = 0.015 and in ER positive patients: C) OS: 53 low vs. 35 high, log rank p = 0.017; D) DMFS: 49 low vs. 33 high, log rank p = 0.029.

Survival was compared between patients with tumours that expressed high mRNA levels (above average) of *TOX3* or *LOC643714* as opposed to low levels (below average). Patients with tumours that express high *TOX3* mRNA had shorter DMFS and OS (p = 0.015 and p = 0.022, respectively, Figure 4). This effect was independent of lymph node status. Survival analysis within subgroups depending on ER status revealed that high *TOX3* mRNA expression in patients with ER positive tumours resulted in shorter DMFS and OS than in patients with low *TOX3* mRNA (p = 0.029 and p = 0.017, respectively, Figure 4). This effect was mostly due to tumours of the luminal B subtype because their removal from the analysis resulted in loss of the effect of *TOX3* mRNA on OS and DMFS (log rank p = 0.64 and log rank p = 0.86, respectively) whereas removal of luminal A tumours had small effect on survival (OS: log rank p = 0.02 and DMFS: log rank p = 0.05; Additional file 1: Figure S4). Fewer tumours are within the ER negative group and the effect of *TOX3* mRNA level on survival was not statistically significant. There was no effect of high *LOC643714* mRNA on DMFS or OS.

## Discussion

In summary, the minor allele of SNP rs3803662 was associated with shorter survival in breast cancer patients with luminal A tumours, with lower mRNA expression of *TOX3* in ER positive tumours as well as being more frequently observed in lobular tumours (Additional file 1: Figure S5). Also, low expression of *TOX3* and the neighbouring gene *LOC643714* was associated with high Ki67 and the basal tumour subtype while high mRNA expression was associated with ER status, positive lymph nodes at diagnosis and shorter survival (Additional file 1: Figure S6).

The risk allele of rs3803662 has been shown to associate with ER and PgR status [6,11,20-22,40], grade of tumours [21,22,40] and diagnosis before the age of 60 [9]. In our study cohort, that was smaller than the aforementioned studies, we did not observe a correlation between genotype at rs3803662 and age or tumour characteristics except that the risk allele was more frequent in patients with lobular tumours, which is in agreement with published data [6,21]. The Breast Cancer Association Consortium (BCAC) recently reported that ER positive patients, homozygous for the risk allele, had poorer overall and breast cancer specific survival than patients, homo- and heterozygous for the non-risk allele [40]. The BCAC study did not include information on the molecular subtypes of the tumours. In our cohort, the risk allele was significantly associated with poor overall survival only in patients with luminal A tumours. The rs3803662 genotype has been shown to associate most strongly with the ER/PgR positive and ERBB2 negative subgroups [21], which is the expression pattern of tumours of the luminal A subtype, further supporting that the effect seen in the BCAC study may be connected to luminal A tumours.

A decrease in *TOX3* mRNA according to the rs3803662 genotype was observed in a large Dutch study of 1401 breast tumours [33] but analysis according to ER status was not reported. In our study, the allele-dependent decrease of *TOX3* or *LOC643714* mRNA level was only observed in ER positive tumours. The *TOX3* mRNA levels were quantified by microarrays and confirmed by qRT-PCR. A significant association was not seen using the latter method, possibly due to the lower number of tumours that were available for qRT-PCR. Expression of *LOC643714* was only measured by qRT-PCR and a positive correlation was found between *TOX3* and *LOC643714* mRNA levels. The closely located genes are potentially co-regulated. *LOC643714* mRNA expression in breast tumours was very low and near the detection limit of the assay. This was previously observed by Udler et al. who reported negligible mRNA levels of *LOC643714* in normal breast and breast tumours [13]. A larger cohort will be necessary to confirm the association between rs3803662 and *LOC643714* expression.

The rs3803662 genotype may only affect expression of *TOX3* and *LOC643714* in certain tumour subtypes. Although we observed a reduction in gene expression in ER positive tumours, our preliminary analysis suggests that this is limited to luminal A tumours (Additional file 1: Figure S3) and another mechanism may be responsible for controlling expression of these genes in luminal B tumours. Although a higher number of tumours are required to confirm this observation, the majority of the adverse effect of high *TOX3* expression in ER positive tumours was related to luminal B tumours (Additional file 1: Figure S4). Furthermore, higher *TOX3* mRNA was observed in patients with luminal B tumours with metastasis (p = 0.056, data not shown) but more tumours are necessary to observe a significant association of metastasis to bone as has been reported with high *TOX3* expression [34]. The risk allele of rs3803662 was highly correlated with a subgroup of basal tumours that expresses *EGFR* and *CK5/6* but not ER, PgR and ERBB2 [40]. Expression of *TOX3* and *LOC643714* was significantly lower in basal tumours in our cohort but we did not observe differences in expression according to genotype or a difference in allele distribution compared to the other tumour subtypes as has been reported for rs9940048, which is located within *TOX3*[41].

The rs3803662 SNP may regulate expression of genes more distantly located than *TOX3* and *LOC643714*. It lies within an evolutionary conserved region that has an open chromatin conformation suggestive of an active transcription regulatory region [13]. Our preliminary analysis of the association of the rs3803662 risk allele with the expression of genes located within 1 Mb of the SNP revealed an association with the retinoblastoma-like 2 gene (*RBL2*) only, where an increase in expression was seen with the number of risk alleles (p = 0.02, data not shown). This result is in agreement with Udler et al., who reported a dose-dependent association between the rs3803662 risk allele with increased expression of *RBL2* mRNA expression in lymphocytes; however, an association between the rs3803662 genotype and *RBL2* mRNA expression in 77 breast tumours was not seen [13]. Expression of *RBL2* was recently reported to be associated with prognosis [40]. It is a member of the retinoblastoma gene family [42] and has been shown to be involved in cell cycle regulation, and it is frequently deleted in breast tumours [43].

Very low expression of *TOX3* and *LOC643714* was observed in the basal subtype, an observation that has been reported for *TOX3*[41]. The significantly lower *TOX3* and *LOC643714* expression in tumours expressing high Ki67 was due to the relatively high number of basal tumours expressing high Ki67. Expression correlated significantly with metastasis in ER negative tumours but not with metastasis to bone [34]. High expression of both *TOX3* and *LOC643714* mRNA was observed in tumours of lymph node positive patients but only *TOX3* mRNA correlated with survival where high levels were seen in patients with shorter overall and distant metastasis free survival in the entire group and for patients with ER positive tumours. This discrepancy between *TOX3* and *LOC643714* may possibly be due to fewer *LOC643714* tumours than for *TOX3*, different distribution of available samples into subtypes, or the fact that there was not a correlation between *TOX3* and *LOC643714* in ER positive tumours. It was recently reported that *TOX3* expression was not associated with prognosis [40]. The study was large but the data came from 9 separate studies in publicly available databases. Thus, we suggest that the effect potentially vanished due to variability in study design and data acquisition.

The biological mechanism that potentially explains our results is the implication of *TOX3* in proliferation and apoptosis. Knockdown of *TOX3* expression with siRNA in the luminal A breast cancer cell line ZR-75-1 increased cellular proliferation [44]. The same study described a FOXA1 binding site at breast cancer SNP rs4784227, whose risk allele segregates with the risk allele of rs3803662; FOXA1 preferentially bound the site that included the rs4784227 risk allele resulting in a 5-fold decrease in *TOX3* expression [44]. Therefore, the correlation of FOXA1 expression with luminal A tumours [45] could explain lower expression of TOX3 in our study and poorer survival of carriers of the rs3803662 risk allele and luminal A tumours. In these tumours, *TOX3* would appear to be a tumour suppressor. However, a different scenario has been described in neuronal cells that potentially may explain the effect of high *TOX3* mRNA in ER positive tumours which we ascribe to luminal B tumours. Increased expression of *TOX3* protected neuronal cells from cell death by altering expression levels of genes that affect apoptosis [30]. Such function in ER positive breast cancer cells, where *TOX3* expression is high, could promote tumour enlargement, invasion of nearby tissues, seeding of lymph nodes which would subsequently shorten the time until death or distant metastasis. The effect of *TOX3* on apoptosis in neuronal cells was mediated via its interaction with CITED1 and ERE [30] and may be the case for proliferation as well. However, the TOX3 ERE-mediated transcription appeared independent of ER and oestrogen [30] unlike that of CITED1, a co-activator of ER that modulates ER responsive genes [31]. We can only speculate that *LOC643714* has a role in tumour development based on results similar to *TOX3* but its mRNA is not translated and its function is unknown.

Although we assume that mRNA expression of our candidate genes may differently affect breast tumour subtypes, the study is small and the analyses according to ER status or molecular subtypes further reduced the number of tumours. As such, the study is exploratory and the results should be confirmed with a larger number of tumours. Our findings provide a suitable basis for further validation studies.

## Conclusions

Taken together, the data suggest that the rs3803662 genotype may affect expression of *TOX3* and *LOC643714* in tumours that originate in some developmental structures within the breast, such as the lobes, and in different molecular subtypes of tumours, such as luminal A and possibly in subgroups of basal tumours. Furthermore, in other types of breast cancer, possibly luminal B tumours, expression of these genes may be controlled by other mechanisms where high expression has adverse effects on prognosis. Although further analysis is needed, we conclude that genotype at rs3803662 and expression of *TOX3* and *LOC643714* have adverse effect on breast cancer prognosis.

## Abbreviations

*BRCA1*: Breast cancer 1; *BRCA2*: Breast cancer 2; *CITED1*: Cbp/p300-interacting transactivator, with Glu/Asp-rich carboxy-terminal domain, 1; *DMFS*: Distant metastasis-free survival; *ERBB2*: V-erb-b2-erythroblastic leukemia viral oncogene homolog 2; *ER*: Oestrogen receptor; *LD*: Linkage disequilibrium; *OS*: Overall survival; *PgR*: Progesterone receptor; *RBL2*: Retinoblastoma-like 2; *SNP*: Single nucleotide polymorphism; *TOX3*: TOX high mobility group box family member 3.

## Competing interests

The authors declare that they have no competing interests.

## Authors’ contributions

ETG performed the RT-PCR experiments, contributed to data analysis as well as the writing of the manuscript. RBB conceived the study, interpreted the data and contributed to the writing of the manuscript. AA participated in the microarray experiments and critically revised the manuscript. LTA interpreted the data and critically reviewed the manuscript. HG performed the genotyping, participated in the microarray experiments and analyzed the data. BAA participated in the acquisition and interpretation of the pathologic data and critical reviewing of the manuscript. OTJ participated in acquisition of the clinical data and critical reviewing of the manuscript. IR analysed and interpreted the data, and led the writing of the manuscript. All authors read and approved the final manuscript.

## Pre-publication history

The pre-publication history for this paper can be accessed here:

http://www.biomedcentral.com/1471-2407/12/621/prepub

## Supplementary Material

Additional file 1 **Figures S1 – S6.** Supplementary Figure S1. Comparison between DMFS in patients with luminal A tumours carrying the minor vs. major rs3803662 allele. Supplementary Figure S2. Correlation of the rs3803662 genotype with expression levels of *TOX3* and *LOC643714*. Supplementary Figure S3. Correlation of the rs3803662 genotype with expression levels of *TOX3* and *LOC643714* in luminal tumours. Supplementary Figure S4. Patients with luminal B tumours contribute the majority of the adverse effect correlated with high TOX3 in ER positive tumours. Supplementary Figure S5. A schematic of the correlation analyses of the rs3803662 genotype with clinical and tumour characteristics. Supplementary Figure S6. A schematic of the correlation analyses of the mRNAs of *TOX3* and *LOC64371* with clinical and tumour characteristics. Click here for file

Additional file 2 **Tables S1 – S3.** Supplementary Table S1. Association between the SNP rs3803662 genotype and clinical and pathological parameters (*n =* 160). Supplementary Table S2. Correlation between *TOX3* and *LOC643714* mRNA levels in ER positive tumours with breast cancer clinical and pathological characteristics. Supplementary Table S3. *TOX3* and *LOC643714* mRNA expression in ER negative tumour samples compared with breast cancer clinical and pathological characteristics. Click here for file
