# Supplementary material for: The risk allele of SNP rs3803662 and the mRNA level of its closest genes TOX3 and LOC643714 predict adverse outcome for breast cancer patients
Source: BMC Cancer. 2012 Dec 27;12:621. doi: 10.1186/1471-2407-12-621 (PMC3553017; doi:10.1186/1471-2407-12-621)
Supplement: Additional file 1 — Figures S1 – S6. Supplementary Figure S1. Comparison between DMFS in patients with luminal A tumours carrying the minor vs. major rs3803662 allele. Supplementary Figure S2. Correlation of the rs3803662 genotype with expression levels of TOX3 and LOC643714. Supplementary Figure S3. Correlation of the rs3803662 genotype with expression levels of TOX3 and LOC643714 in luminal tumours. Supplementary Figure S4. Patients with luminal B tumours contribute the majority of the adverse effect correlated with high TOX3 in ER positive tumours. Supplementary Figure S5. A schematic of the correlation analyses of the rs3803662 genotype with clinical and tumour characteristics. Supplementary Figure S6. A schematic of the correlation analyses of the mRNAs of TOX3 and LOC64371 with clinical and tumour characteristics. [file 1471-2407-12-621-S1.docx]

**Supplementary Figure S1. Comparison between DMFS in patients with luminal A tumours carrying the minor vs. major rs3803662 allele.** DMFS was compared in patients with luminal A tumours who were heterozygous or homozygous for the minor allele (CT/TT, n = 18) as opposed to homozygous for the major allele (CC, n = 24), log rank p = 0.24.

**Supplementary Figure S2. Correlation of the rs3803662 genotype with expression levels of *TOX3* and *LOC643714*.** mRNA levels of *TOX3* (**A**) and *LOC643714* (**B**) were quantified with qRT-PCR in 139 and 126 breast tumours, respectively. The rs3803662 genotype was correlated with the continuous log_2_ transformed mRNA values of *TOX3* (p = 0.11) and *LOC643714* (p = 0.24). ANOVA was used to test for differences between the genotype categories; the T allele is the risk allele of rs3803662.

**Supplementary Figure S3. Correlation of the rs3803662 genotype with expression levels of *TOX3* and *LOC643714* in luminal tumours.**  Correlation of TOX3 and LOC643714 mRNA in luminal A (top) and luminal B (bottom) tumours. The log_2_ transformed mRNA values were used as continuous variables to calculate the correlation with the rs3803662 genotype using an ANOVA. In luminal A tumours: (**A**) TOX3, p = 0.14 and (**B**) LOC643714, p = 0.26 and in luminal B tumours: (**C**) TOX3, p = 0.84 and (**D**) LOC643714, p = 0.50.

**Supplementary Figure S4. Patients with luminal B tumours contribute the majority of the adverse effect correlated with high TOX3 in ER positive tumours.** The effect of luminal A and luminal B tumours on survival was analysed by removing patients carrying (left) luminal A or (right) luminal B tumours from the (top) OS and (bottom) DMFS analyses. ER positive patients remaining in the OS analyses were (**A**) 25 with low and 23 with high TOX3 mRNA (log rank p = 0.02) and (**B**) 42 with low and 15 with high TOX3 mRNA (log rank p = 0.64). ER positive patients remaining in the DMFS analyses were (**C**) 24 with low and 22 with high TOX3 mRNA (log rank p = 0.05) and (**D**) 39 with low and 14 with high TOX3 mRNA (log rank p = 0.86).


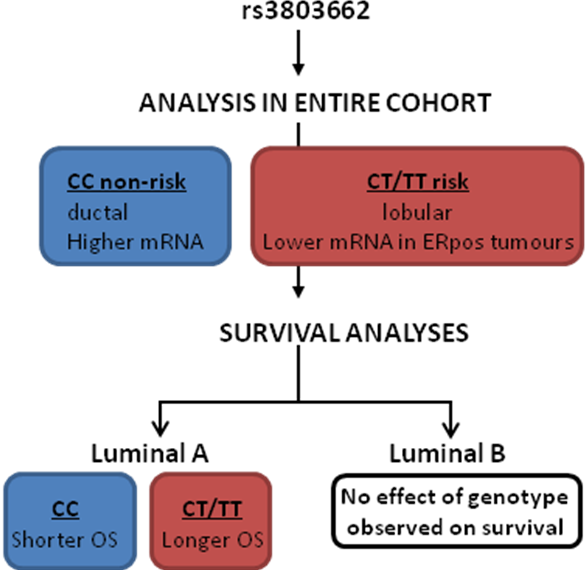


**Supplementary Figure S5. A schematic of the correlation analyses of the rs3803662 genotype with clinical and tumour characteristics.** A correlation between the genotypes at rs3803662 and the clinical and tumour characteristics was performed in the entire cohort. The homozygous (TT) and heterozygous (CT) carriers of the risk allele were grouped together in the analyses. Due to the allele-dependent expression of *TOX3* in ER positive tumours the survival analysis was performed separately in luminal A and B tumours. The genotype did not affect survival in the entire cohort or the ER positive group. Only significant results (p < 0.5) are depicted in the scheme, blue for the homozygous non-risk allele versus red for the homo- and heterozygous risk allele.


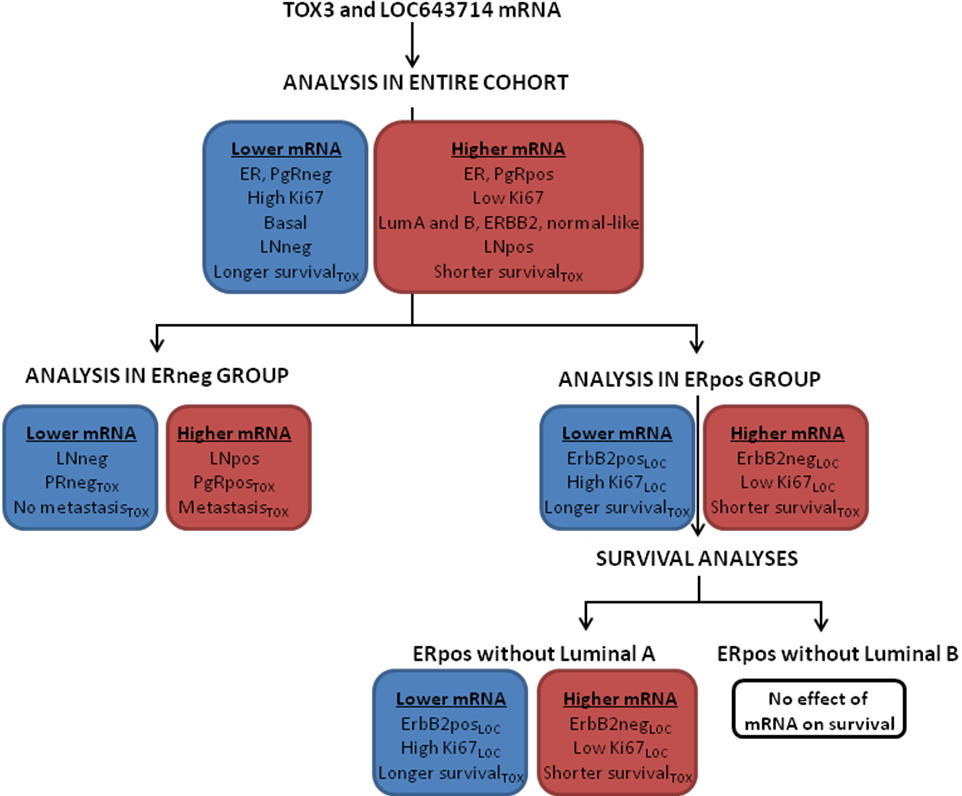


**Supplementary Figure S6. A schematic of the correlation analyses of the mRNAs of *TOX3* and *LOC64371* with clinical and tumour characteristics.** A correlation was performed between expression of *TOX3* or *LOC643714* and clinical and tumour characteristics in the entire cohort. Based on the results, the analyses were repeated in ER positive and negative tumours. Subsequently, the survival analyses were repeated after the removal of either luminal A or luminal B tumours. Only significant results (p < 0.5) are depicted in the scheme, blue for mRNA expression below average and red expression above average. If a correlation was observed for one gene only it is indicated by TOX or LOC as a subscript.
