# Supplementary material for: The risk allele of SNP rs3803662 and the mRNA level of its closest genes TOX3 and LOC643714 predict adverse outcome for breast cancer patients
Source: BMC Cancer. 2012 Dec 27;12:621. doi: 10.1186/1471-2407-12-621 (PMC3553017; doi:10.1186/1471-2407-12-621)
Supplement: Additional file 2 — Tables S1 – S3. Supplementary Table S1. Association between the SNP rs3803662 genotype and clinical and pathological parameters (n = 160). Supplementary Table S2. Correlation between TOX3 and LOC643714 mRNA levels in ER positive tumours with breast cancer clinical and pathological characteristics. Supplementary Table S3. TOX3 and LOC643714 mRNA expression in ER negative tumour samples compared with breast cancer clinical and pathological characteristics. [file 1471-2407-12-621-S2.docx]

**Supplementary Table S1.** Association between the SNP rs3803662 genotype and clinical and pathological parameters (*n =* 160).

|  |  |  | | **CC** | **CT or TT** |  |
| --- | --- | --- | --- | --- | --- | --- |
|  |  | *n* | | 90 | 70 | p-value |
| **Age (years)** | | | |  |  |  |
|  | < 50 |  | | 41 (46%) | 25 (36%) | 0.26 |
|  | ≥ 50 |  | | 49 ((54%) | 45 (64%) |  |
| **Tumour size (mm)** | | | |  |  |  |
|  | ≤ 20 |  | | 27 (31%) | 22 (32%) | 1 |
|  | > 20 |  | | 60 (69%) | 47 (68%) |  |
|  | unknown |  | | 3 | 1 |  |
| **Estrogen receptor** | | | |  |  |  |
|  | negative |  | | 33 (38%) | 20 (29%) | 0.24 |
|  | positive |  | | 53 (62%) | 49 (71%) |  |
|  | unknown |  | | 4 | 1 |  |
| **Progestrerone receptor** | | | |  |  |  |
|  | negative |  | | 41 (48%) | 31 (46%) | 0.87 |
|  | positive |  | | 44 (52%) | 36 (54%) |  |
|  | unknown |  | | 5 | 3 |  |
| **ErbB2 status** | | | |  |  |  |
|  | negative |  | | 73 (82%) | 58 (83%) | 1 |
|  | positive |  | | 16 (18%) | 12 (17%) |  |
|  | unknown |  | | 1 | 0 |  |
| **Nodal status** | | | |  |  |  |
|  | negative |  | | 36 (45%) | 27 (42%) | 0.74 |
|  | positive |  | | 44 (55%) | 38 (58%) |  |
|  | unknown |  | | 10 | 5 |  |
| **Ki 67** | | | |  |  |  |
|  | Low |  | | 60 (68%) | 52 (75%) | 0.38 |
|  | High |  | | 28 (32%) | 17 (25%) |  |
|  | unknown |  | | 2 | 1 |  |
| **Histograde** | | | |  |  |  |
|  | 1 |  | | 8 ((9%) | 8 (11%) | 0.89 |
|  | 2 |  | | 49 (56%) | 39 (56%) |  |
|  | 3 |  | | 31 (35%) | 23 (33%) |  |
|  | unknown |  | | 2 | 0 |  |
| **Familial status ^1^** | | | |  |  |  |
|  | BRCA 2 | |  | 15 (17%) | 14 (20%) | 0.26 |
|  | BRCAx | |  | 41 (47%) | 37 (53%) |  |
|  | Sporadic | |  | 31 (36%) | 19 (27%) |  |
|  | Unknown | |  | 3 | 0 |  |
| **Subtype^2^** | | | |  |  |  |
|  | Basal | |  | 17 (22%) | 8 (13%) | 0.24 |
|  | ERBB2 |  | | 10 (13%) | 7 (12%) |  |
|  | Luminal A |  | | 28 (36%) | 20 (33%) |  |
|  | Luminal B |  | | 17 (22%) | 14 (23%) |  |
|  | Normal-like |  | | 5 (7%) | 11 (18%) |  |
|  | Unclass/unknown |  | | 13 | 10 |  |
| **Histological type** | | | |  |  |  |
|  | Ductal |  | | 78 (92%) | 54 (77%) | 0.04 |
|  | Lobular |  | | 3 (4%) | 8 (11.5%) |  |
|  | other |  | | 4 (5%) | 8 (11.5%) |  |
|  | unknown |  | | 5 | 0 |  |
| **Metastasis** | |  | |  |  |  |
|  | negative |  | | 36 (47%) | 36 (55%) | 0.24 |
|  | positive |  | | 40 (53%) | 29 (45%) |  |
|  | unknown |  | | 14 | 5 |  |

All *p* values were calculated with Fisher’s exact test.

^1^ One tumour was BRCA1 positive and was not used in the familial status calculations.

^2^ Unclassified/unknown samples were not included in the statistical calculation.

**Supplementary Table S2.** Correlation between TOX3 and LOC643714 mRNA levels in ER positive tumours with breast cancer clinical and pathological characteristics.

|  |  |  | ***TOX3* mRNA** |  |  | ***LOC643714* mRNA** |  |
| --- | --- | --- | --- | --- | --- | --- | --- |
|  |  | n=91 | median (25% and 75%) | p-value | n=81 | median (25% and 75%) | p-value |
| **Age** |  |  |  |  |  |  |  |
|  | < 50 | 32 (35%) | 2.1 (1.1 - 4.8) | 0.69 | 28 (35%) | 1.3 E-3 (2.6 E-4 - 3.6 E-2) | 0.44 |
|  | ≥ 50 | 59 (65%) | 2.1 (1.1 - 4.3) |  | 53 (65%) | 3.2 E-3 (6.4 E-4 - 0.18) |  |
| **Tumour size (mm)** | |  |  |  |  |  |  |
|  | ≤ 20 | 29 (32%) | 1.4 (1.1 - 3.6) | 0.17 | 26 (32%) | 3.3 E-3 (3.8 E-4 - 1.4 E-2) | 0.20 |
|  | > 20 | 61 (68%) | 2.4 (1.1 - 5.2) |  | 54 (68%) | 2.3 E-3 (4.7 E-4 - 0.36) |  |
|  | unknown | 1 |  |  | 1 |  |  |
| **Progestrerone receptor** | |  |  |  |  |  |  |
|  | negative | 27 (31%) | 3.6 (1.0 - 5.1) | 0.65 | 22 (29%) | 1.2 E-3 (3.6 E-4 - 0.05) | 0.11 |
|  | positive | 59 (69%) | 2.1 (1.2 - 4.3) |  | 54 (71%) | 3.4 E-3 (5.1 E-4 - 0.5) |  |
|  | unknown | 5 |  |  | 5 |  |  |
| **ErbB2 status** | |  |  |  |  |  |  |
|  | negative | 81 (90%) | 2.2 (1.1 - 4.4) | 0.73 | 72 (90%) | 3.4 E-3 (5.3 E-4 - 0.14) | 0.01 |
|  | positive | 9 (10%) | 1.6 (0.8 - 5.6) |  | 8 (10%) | 4.6 E-4 (2.5 E-4 - 1.1 E-3) |  |
|  | unknown | 1 |  |  | 1 |  |  |
| **Nodal status** | |  |  |  |  |  |  |
|  | negative | 36 (43%) | 1.7 (0.9 - 3.9) | 0.07 | 31 (74%) | 3.2 E-3 (2.6 E-4 - 0.1) | 0.76 |
|  | positive | 47 (57%) | 2.4 (1.3 - 5.3) |  | 43 (26%) | 2.4 E-3 (7.2 E-4 - 0.1) |  |
|  | unknown | 8 |  |  | 7 |  |  |
| **Ki 67** | |  |  |  |  |  |  |
|  | Low | 68 (76%) | 2.2 (1.1 - 4.7) | 0.14 | 63 (79%) | 4.0 E-3 (6.3 E-4 - 0.1) | 0.02 |
|  | High | 21 (24%) | 1.3 (1.0 - 2.4) |  | 17 (21%) | 6.4 E-4 (6.3 E-6 - 1.8 E-3 ) |  |
|  | unknown | 2 |  |  | 1 |  |  |
| **Histograde** | |  |  |  |  |  |  |
|  | 1 | 11 (12%) | 1.1 (0.8 - 3.6) | 0.18 | 11 (14%) | 1.2 E-3 (3.9 E-4 - 5.5 E-2) | 0.46 |
|  | 2 | 58 (65%) | 2.1 (1.1 - 4.7) |  | 49 (62%) | 7.8 E-3 (6.4 E-4 - 0.2) |  |
|  | 3 | 20 (23%) | 3.5 (1.2 - 4.8) |  | 19 (24%) | 9.7 E-4 (2.6 E-4 - 9.0 E-3) |  |
|  | unknown | 2 |  |  | 2 |  |  |
| **Familial status** | |  |  |  |  |  |  |
|  | BRCA 2 | 20 (22%) | 2.9 (1.1 - 5.2) | 0.81 | 18 (23%) | 1.0 E-3 (3.3 E-4 - 3.6 E-3) | 0.13 |
|  | non BRCA 2 | 69 (78%) | 2.1 (1.1 - 4.4) |  | 61 (77%) | 6.4 E-3 (5.3 E-4 - 0.1) |  |
|  | unknown | 2 |  |  | 2 |  |  |
| **Subtype^1^** | |  |  |  |  |  |  |
|  | Basal | 1 (1%) | 0.83 | 0.39 | 1 (1%) | 2.1 E-4 | 0.16 |
|  | ERBB2 | 4 (5%) | 1.4 (1.2 – 2.6) |  | 3 (4%) | 1.8 E-3 (1.1 E-3 – 8.3 E-3) |  |
|  | Luminal A | 38 (48%) | 1.7 (1.1 - 3.5) |  | 33 (47%) | 1.0 E-2 (1.0 E-3 – 1.8 E-1) |  |
|  | Luminal B | 28 (35%) | 3.9 (1.4 - 5.2) |  | 26 (37%) | 1.1 E-3 (6.5 E-4 – 1.7 E-1) |  |
|  | Normal | 9 (11%) | 1.4 (1.3 - 2.4) |  | 7 (10%) | 5.3 E-4 (6.2 E-5 - 1.9 E-3) |  |
|  | Unclass/unknown | 11 |  |  | 11 |  |  |
| **Histological type** | |  |  |  |  |  |  |
|  | Ductal | 73 (83%) | 2.9 (1.1 - 5.2) | 0.28 | 66 (85%) | 3.3 E-3 (5.4 E-4 - 0.2) | 0.50 |
|  | Lobular | 10 (11%) | 1.3 (1.3 - 2.2) |  | 8 (10%) | 1.0 E-3 (4.3 E-4 - 2.2 E-2) |  |
|  | other | 5 (6%) | 1.0 (1.1 - 1.5) |  | 4 (5%) | 4.3 E-2 (5.3 E-4 - 3.6) |  |
|  | unknown | 3 |  |  | 3 |  |  |
| **Metastasis** | |  |  |  |  |  |  |
|  | negative | 42 (51%) | 2.1 (1.1 - 3.6) | 0.22 | 40 (55%) | 1.6 E-3 (4.4 E-4 - 7.5 E-2) | 0.16 |
|  | positive | 40 (49%) | 2.2 (1.1 - 5.2) |  | 33 (45%) | 6.4 E-3 (6.7 E-4 - 0.2) |  |
|  | unknown | 9 |  |  | 8 |  |  |

The table shows the median and the 25^th^ and 75^th^ percentiles. The *p-* value is calculated using log2 transformed data with a *t-*test or ANOVA.

^1^ The 1 basal sample and the unclassified samples were not used in the statistical calculations.

**Supplementary Table S3.** *TOX3* and *LOC643714* mRNA expression in ER negative tumour samples compared with breast cancer clinical and pathological characteristics.

|  |  | **n** | **TOX3 mRNA** | **p-value** | **n** | **LOC643714 mRNA** | **p-value** |
| --- | --- | --- | --- | --- | --- | --- | --- |
|  |  | *44* | median (25% and 75%) |  | *41* | median (25% and 75%) |  |
| **Age** |  |  |  |  |  |  |  |
|  | < 50 | 24 (55%) | 1.5 (0.3 - 4.3) | 0.59 | 22 (54%) | 4.5 E-6 (6.7 E-7 - 2.7 E-4) | 0.36 |
|  | ≥ 50 | 20 (45%) | 1.3 (0.8 - 1.8) |  | 19 (46%) | 7.1 E-5 (1.1 E-6 - 2.7 E-3) |  |
| **Tumour size (mm)** | |  |  |  |  |  |  |
|  | ≤ 20 | 11 (26%) | 1.4 (0.3 - 3.2) | 0.58 | 11 (27%) | 5.9 E-7 (5.9 E-7 - 1.2 E-4) | 0.31 |
|  | > 20 | 32 (74%) | 1.3 (0.6 - 3.3) |  | 29 (73%) | 3.1 E-6 (3.1 E-6 - 1.2 E-3) |  |
|  | unknown | 1 |  |  | 1 |  |  |
| **Progestrerone receptor** | |  |  |  |  |  |  |
|  | negative | 37 (86%) | 1.1 (0.2 - 2.2) | 0.002 | 36 (90%) | 6.1 E-6 (5.0 E-7 - 2.9 E-4) | 0.24 |
|  | positive | 6 (14%) | 2.0 (1.8 - 3.7) |  | 4 (10%) | 2.6 E-2 (2.4 E-4 - 0.3) |  |
|  | unknown | 1 |  |  | 1 |  |  |
| **ErbB2 status** | |  |  |  |  |  |  |
|  | negative | 29 (66%) | 1.2 (0.2 - 1.9) | 0.20 | 31 (76%) | 6.3 E-6 (3.1 E-7 - 3.8 E-4) | 0.42 |
|  | positive | 15 (34%) | 2.2 (0.8 - 4.7) |  | 10 (24%) | 1.8 E-4 (2.6 E-6 - 8.6 E-4) |  |
| **Nodal status** | |  |  |  |  |  |  |
|  | negative | 19 (46%) | 0.3 (0.1 - 1.3) | < 0.001 | 20 (54%) | 2.2 E-6 (2.0 E-7 - 1.3 E-4) | 0.01 |
|  | positive | 22 (54%) | 2.0 (1.2 - 4.7) |  | 17 (46%) | 3.1 E-4 (9.3 E-6 - 2.7 E-2) |  |
|  | unknown | 3 |  |  | 4 |  |  |
| **Ki 67** | |  |  |  |  |  |  |
|  | Low | 26 (60%) | 1.4 (0.7 - 2.9) | 0.24 | 21 (53%) | 9.3 E-6 (6.4 E-7 - 4.8 E-3) | 0.42 |
|  | High | 17 (40%) | 0.8 ( 0.2 - 1.9) |  | 19 (47%) | 6.3 E-6 (9.7 E-7 - 1.7 E-4) |  |
|  | unknown | 1 |  |  | 1 |  |  |
| **Histograde** | |  |  |  |  |  |  |
|  | 1-2 | 17 (39%) | 1.4 (1.1 - 2.2) | 0.19 | 17 (41%) | 7.1 E-5(1.2 E-6 - 9.7 E-4) | 0.53 |
|  | 3 | 27 (61%) | 0.9 (0.2 - 3.5) |  | 24 (59%) | 6.0 E-6(5.0 E-7 - 2.9 E-4) |  |
| **Familial status ^1^** | |  |  |  |  |  |  |
|  | BRCA 2 | 5 (12%) | 0.3 (0.2 - 3.8) | 0.42 | 6 (15%) | 3.4 E-6(4.1 E-7 - 2.3 E-3) | 0.52 |
|  | non BRCA 2 | 38 (88%) | 1.4 (0.6 - 2.9) |  | 34 (85%) | 5.4 E-5(1.3 E-6 - 8.7 E-4) |  |
|  | unknown | 1 |  |  | 1 |  |  |
| **Subtype^2^** | |  |  |  |  |  |  |
|  | Basal | 19 (51%) | 0.4 (0.1 - 1.3) | 0.01 | 22 (63%) | 2.2 E-6 (2.2 E-7 - 1.6 E-4) | 0.06 |
|  | ERBB2 | 11 (30%) | 2.2 (1.1 - 5.3) |  | 6 (17%) | 7.6 E-4 (2.7 E-4 - 2.0 E-2) |  |
|  | Luminal A | 3 (8%) | 1.8 (1.5 - 1.9) |  | 3 (9%) | 9.3 E-6 (5.0 E-6 - 2.6 E-2) |  |
|  | Luminal B | 1 (3%) | 10.1 |  | 1 (3%) | 0.6 |  |
|  | Normal | 3 (8%) | 3.8 (2.0 – 5.0) |  | 3 (9%) | 1.3 E-3 (6.5 E-4 – 3.4 E-2) |  |
|  | Unclass/unknown | 7 |  |  | 6 |  |  |
| **Histological type** | |  |  |  |  |  |  |
|  | Ductal | 39 (91%) | 1.4 (0.5 - 4.0) | 0.29 | 35 (88%) | 6.0 E-5 (5.9 E-7 - 1.1 E-3) | 0.02 |
|  | other | 4 (9%) | 0.7 (0.1 - 1.3) |  | 4 (12%) | 5.8 E-6 (3.3 E-6 - 6.3 E-6) |  |
|  | unknown | 1 |  |  | 1 |  |  |
| **Metastasis** | |  |  |  |  |  |  |
|  | negative | 18 (44%) | 0.6 (0.2 - 1.8) | 0.015 | 20 (51%) | 6.0 E-6 (5.7 E-7 - 2.0 E-4) | 0.27 |
|  | positive | 23 (56%) | 1.4 (0.9 - 4.0) |  | 19 (49%) | 4.8 E-5 (1.4 E-6 - 1.4 E-2) |  |
|  | unknown | 3 |  |  | 2 |  |  |

The table shows the median and the 25^th^ and 75^th^ percentiles. The - value is calculated on log2 transformed data with a *t-*test and ANOVA.

^1^ One tumour sample was BRCA1 positive and was not used in familial status calculations.

^2^ The difference between TOX3 mRNA in basal and ERBB2 tumours was significant, p = 0.02.
